# DARPP-32: from neurotransmission to cancer

**DOI:** 10.18632/oncotarget.7268

**Published:** 2016-02-08

**Authors:** Abbes Belkhiri, Shoumin Zhu, Wael El-Rifai

**Affiliations:** ^1^ Department of Surgery, Cancer Biology, and Vanderbilt-Ingram Cancer Center, Vanderbilt University Medical Center, Nashville, TN, USA; ^2^ Department of Veterans Affairs, Tennessee Valley Healthcare System, Nashville, TN, USA

**Keywords:** DARPP-32, t-DARPP, PPP1R1B, neurotransmission, cancer

## Abstract

Dopamine and cAMP-regulated phosphoprotein Mr 32,000 (DARPP-32), also known as phosphoprotein phosphatase-1 regulatory subunit 1B (PPP1R1B), was initially discovered as a substrate of dopamine-activated protein kinase A (PKA) in the neostriatum in the brain. While phosphorylation at Thr-34 by PKA converts DARPP-32 into a potent inhibitor of protein phosphatase 1 (PP1), phosphorylation at Thr-75 transforms DARPP-32 into an inhibitor of PKA. Through regulation of DARPP-32 phosphorylation and modulation of protein phosphatase and kinase activities, DARPP-32 plays a critical role in mediating the biochemical, electrophysiological, and behavioral effects controlled by dopamine and other neurotransmitters in response to drugs of abuse and psychostimulants. Altered expression of DARPP-32 and its truncated isoform (t-DARPP), specifically in the prefrontal cortex, has been associated with schizophrenia and bipolar disorder. Moreover, cleavage of DARPP-32 by calpain has been implicated in Alzheimer's disease. Amplification of the genomic locus of DARPP-32 at 17q12 has been described in several cancers. DARPP-32 and t-DARPP are frequently overexpressed at the mRNA and protein levels in adenocarcinomas of the breast, prostate, colon, and stomach. Several studies demonstrated the pro-survival, pro-invasion, and pro-angiogenic functions of DARPP-32 in cancer. Overexpression of DARPP-32 and t-DARPP also promotes chemotherapeutic drug resistance and cell proliferation in gastric and breast cancers through regulation of pro-oncogenic signal transduction pathways. The expansion of DARPP-32 research from neurotransmission to cancer underscores the broad scope and implication of this protein in disparate human diseases.

## INTRODUCTION

In a study to characterize the mode of action of neurotransmitters in different brain regions, Walaas and colleagues [1] investigated proteins phosphorylated by calcium- and cyclic-AMP (cAMP)-activated protein kinases. Notably, a 32-kDa protein was found abundantly in spiny neurones of the neostriatum. As the phosphorylation of this protein was regulated by dopamine and cAMP, it was named DARPP-32 (dopamine and cAMP-regulated phosphoprotein Mr 32,000) [2]. In addition to the brain, DARPP-32 is expressed in adrenal medulla, kidney, and parathyroid cells [3-5]. Because it was shown to inhibit protein phosphatase-1 (PP1) following phosphorylation of Thr-34 by protein kinase A (PKA) [6], DARPP-32 is also known as phosphoprotein phosphatase-1 regulatory subunit 1B (PPP1R1B). DARPP-32 has been shown to play a key role in mediating the biochemical, electrophysiological, and behavioral effects of dopamine on dopaminoceptive neurons (for reviews, see [7-9]). DARPP-32 has also been implicated in mediating the actions of other neurotransmitter systems such as serotonin and glutamate, in response to a variety of drugs of abuse (for reviews, see [10, 11]). El-Rifai and colleagues discovered frequent amplification of 17q12, the locus of DARPP-32, in gastric and esophageal adenocarcinomas [12, 13]. Subsequently, this group reported that DARPP-32 and its truncated isoform; they cloned and named t-DARPP, are amplified and overexpressed in gastric cancer [14]. This first report led to a series of studies on the role of DARPP-32 and t-DARPP in different biological aspects of gastric cancer and other malignancies. In this review, we briefly outline the role of DARPP-32 in neurotransmission and its recent implication in central nervous system (CNS) disorders, and elaborate on the novel biological functions of DARPP-32 and t-DARPP in human carcinogenesis.

## MODULATION OF DARPP-32 IN NEUROTRANSMISSION AND CNS DISORDERS

Since its discovery three decades ago, DARPP-32 has been shown in a large body of work as a central signaling molecule activated by a diverse array of neurotransmitters such as dopamine, glutamate, serotonin, adenosine, and gamma-aminobutyric acid (GABA) [9, 15, 16]. In response to drugs of abuse and psychostimulants, these neurotransmitters regulate the phosphorylation state of DARPP-32, which converts it to an inhibitor of either a protein phosphatase (PP1) or a protein kinase (PKA). The state of phosphorylation of DARPP-32 is regulated by tonic activation of dopamine D_1_ and D_2_ receptors, and adenosine A_2A_ receptor [17]. DARPP-32 may be phosphorylated at Thr-34 by PKA in distinct subpopulations of medium spiny neurons that express either D_1_ or A_2A_ receptors [18, 19]. Like dopamine, adenosine acts on A_2A_ receptor, using cAMP as a mediator in the process, activating PKA and increasing DARPP-32 phosphorylation at Thr-34 [20]. The D_2_ receptor exists as two different isoforms generated by alternative splicing: the long (D2L) and the short (D2S) [21]. The D2S receptor specifically regulates the state of phosphorylation and activity of tyrosine hydroxylase (TH) in nigrostriatal presynaptic terminals, whereas the D2L receptor is mainly involved in the regulation of DARPP-32 phosphorylation in postsynaptic striatal medium spiny neurons [22]. Activation of the D_2_ receptor decreases DARPP-32 phosphorylation by two different mechanisms. First, in medium spiny neurons that co-express D_1_ and D_2_ classes of dopamine receptors, activation of D_2_ receptors decreases cAMP levels. When D_2_ receptors are co-expressed with adenosine A_2A_ receptor, it can result in a decrease of cAMP levels, decreasing the activity of both PKA and in phosphorylation of DARPP-32 at Thr-34. Second, activation of D_2_ receptor leads to an increase in Ca^2+^ levels and increased activity of protein phosphatase 2B (PP-2B), resulting in an increase in dephosphorylation of DARPP-32 at Thr-34 [23]. In the intact striatum, a blockade of tonic dopamine D_2_ receptor activation increases DARPP-32 phosphorylation at Thr-34 and can be counteracted by blocking either adenosine A_2A_ or dopamine D_1_ receptors [17].

**Figure 1:**
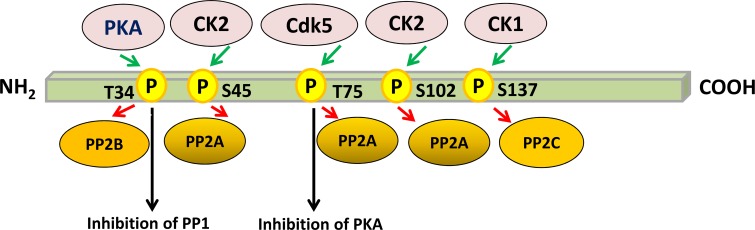
Regulation of PP1 and PKA by multisite phosphorylation of DARPP-32 Phosphorylation of DARPP-32 by protein kinases is indicated by green arrows, whereas dephosphorylation of DARPP-32 by protein phosphatases is depicted by red arrows. Phosphorylation of DARPP-32 at Thr-34 by PKA converts it into a potent inhibitor of PP1. However, phosphorylation of DARPP-32 at Thr-75 by CDK5 transforms it into an inhibitor of PKA. Dephosphorylation of Ser-137 by PP2C facilitates dephosphorylation of Thr-34 by PP2B, thereby removing the PKA-induced inhibition of PP1. Phosphorylation of Ser-45 and Ser-102 has no effect on the potency of DARPP-32 as an inhibitor of PP1. The scheme is based on [29].

The phosphorylation of DARPP-32 at Thr-34 converts it into a potent inhibitor of PP1 [6, 24, 25]. PP1 is involved in regulating the activity of a large number of phosphoproteins, including voltage-dependent sodium and calcium channels, the electrogenic pump Na^+^, K^+^-ATPase, and neurotransmitter receptors [26]. DARPP-32-mediated inhibition of PP1 increases the phosphorylation of neurotransmitter receptors and ion channels crucial for synaptic function and plasticity [9]. Bibb and colleagues reported that DARPP-32 was converted into an inhibitor of PKA when phosphorylated at Thr-75 by cyclin-dependent kinase 5 (Cdk5) [27]. Decreasing phospho-Thr-75 DARPP-32 in striatal slices, either by a Cdk5-specific inhibitor or by using genetically altered mice, increases dopamine-induced phosphorylation of PKA substrates and augments peak voltage-gated calcium currents [28, 29]. This suggests that, depending on which particular amino acid residue is phosphorylated, DARPP-32 can function as either a kinase or a phosphatase inhibitor. This unique dual action appears to be especially important in regulating the efficacy of dopaminergic neurotransmission [8, 27] (Figure 1).

Casein kinase 1 (CK1) can phosphorylate DARPP-32 at Ser-137, which has an important regulatory role because it inhibits the dephosphorylation of Thr-34 by calcineurin. This inhibition takes place only when phospho-Ser-137 and phospho-Thr-34 are present on the same DARPP-32 molecule [30]. Phosphorylation of DARPP-32 by CK1, which is highly active in striatonigral neurons, does not alter its ability to inhibit PP1 [30]. DARPP-32 phosphorylated by CK1 is a substrate for protein phosphatases 2A and 2C. However, in substantia nigra slices, dephosphorylation of Ser-137 was markedly sensitive to decreased temperature, and not significantly affected by the presence of okadaic acid under conditions where dephosphorylation of Thr-34 by protein phosphatase 2A was inhibited. This suggests that, in neurons, phospho-Ser-137 DARPP-32 is dephosphorylated by protein phosphatase 2C, but not 2A. Thus, DARPP-32 appears to be a component of a regulatory cascade of phosphatases in which dephosphorylation of Ser-137 by protein phosphatase 2C facilitates dephosphorylation of Thr-34 by calcineurin, removing the cAMP-induced inhibition of PP1 [31]. DARPP-32 is also phosphorylated on Ser-45 and Ser-102 by casein kinase 2 (CK2) [32]. Phosphorylation by CK2 has no effect on the potency of DARPP-32 as an inhibitor of PP1, which strictly depends on phosphorylation of Thr-34 by cAMP-dependent protein kinase [33] (Figure 1).

Following the discovery of t-DARPP by El-Rifai's group in cancer [14], post-mortem studies on human brains suggest that the expression of t-DARPP transcript is altered in patients with schizophrenia and bipolar disorder specifically in the prefrontal cortex, and the higher t-DARPP expression was associated with worse cognitive performance [34]. Another study indicated a selective reduction in DARPP-32 expression in the prefrontal cortex in patients with schizophrenia, suggesting that altered expression of DARPP-32 may be associated with the development of the disease [35]. Cho and colleagues investigated the role of DARPP-32 in Alzheimer's disease (AD), and found that DARPP-32 is cleaved at Thr-153 by calpain, which reduces the phosphorylation of cAMP-response element-binding protein (CREB), a target of PP1 and important for cognitive function. The cleavage of DARPP-32 induces loss of its inhibitory function on PP1. The data suggest a novel mechanism by which cleavage of DARPP-32 leads to dysregulation of CREB signaling in AD [36].

## IDENTIFICATION AND IMPLICATION OF DARPP-32 IN CANCER

**Figure 2:**
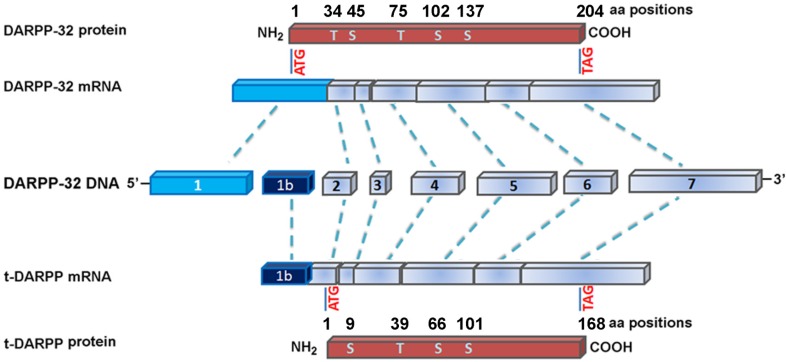
Genomic structure of DARPP-32 and its truncated isoform, t-DARPP DARPP-32 and t-DARPP share identical sequence from exon 2 to the 3′ end. Of note, exon 1 of t-DARPP is spliced from the intron 1 of DARPP-32. DARPP-32 (1,983 bp) encodes 204 amino acids, whereas t-DARPP (1,502 bp) encodes 168-amino-acids protein. The major phosphorylation sites on the proteins are indicated by T, threonine, and S, serine. DARPP-32 contains five phosphorylation sites at T34, S45, T75, S102, and S137, whereas t-DARPP lacks the T34 phosphorylation site of DARPP-32. The scheme is based on [39].

Earlier studies using comparative genomic hybridization have pointed out to frequent amplification at the 17q12 locus in several malignancies including gastric and esophageal cancers [12, 13, 37]. A systematic analysis of copy number and expression levels of the 17q amplicon-specific genes revealed an increase in copy numbers of 18 genes such as *ERBB2*, *TOP2A*, *GRB7*, and expression sequence tag (EST AA552509) in gastric cancers [38]. El-Rifai and colleagues demonstrated that EST AA552509 was frequently amplified and consistently overexpressed at 17q in gastric cancers, and they were first to report that DARPP-32 is the target gene for overexpression of EST AA552509 [14]. Cloning and sequence assembly analyses indicated that EST AA552509 corresponded to the 3′-untranslated region of the DARPP-32 gene. Further analyses of the nearby ESTs, two ESTs (BF725600 and BF724182) were found to overlap with the 5′ end of DARPP-32 exon 1. An additional 467 bp untranslated mRNA sequence of DARPP-32 was identified upstream of the previously known translation start site in exon 1. Additional cloning and sequencing verified the full-length DARPP-32 cDNA sequence (AF464196). A novel transcriptional splice variant of DARPP-32 was cloned from gastric cancer tissues (AY070271) and termed truncated isoform of DARPP (t-DARPP) [14]. The novel molecule, t-DARPP, has a unique alternative first exon located within intron 1 of DARPP-32. While DARPP-32 encodes 204 amino acids protein containing four phosphorylation sites (Thr-34, Thr-75, Ser-102, and Ser-137), t-DARPP encodes 168 amino acids protein lacking the Thr-34 phosphorylation site related to PP1 inhibition but maintains the other three phosphorylation sites (Figure 2). Concomitant overexpression of DARPP-32 and t-DARPP was demonstrated in 68% of gastric cancers [39]. In addition, several studies indicated frequent overexpression of DARPP-32 and t-DARPP in adenocarcinomas of the breast, prostate, and colon [40-43]. These findings suggest that DARPP-32 proteins could be implicated in critical steps in the carcinogenesis cascade. Further studies were prompted to investigate the functional role of DARPP-32 and t-DARPP primarily in gastric cancer and other types of malignancies.

### DARPP-32 in tumorigenesis

A few studies have attempted to examine the role of DARPP-32 in the multistep cascade of gastric carcinogenesis. A study using immunohistochemistry analysis on 533 human gastric cancer tissues indicated that DARPP-32 is overexpressed in early stages of gastric tumorigenesis, suggesting that this molecular event may participate in the transition to intestinal metaplasia and in the progression to adenocarcinoma [44]. Based on the concept that fusion genes, which result from chromosomal rearrangements or abnormal transcription, act as potent oncogenes in many types of cancer [45, 46], Yun and colleagues [47] identified novel gene fusions in human gastric cancer cells and tissues. Notably, the presence of DARPP-32-STARD3 fusion transcript, which appears to be generated by RNA processing without chromosomal rearrangement, was exclusively detected in a subset of gastric cancers (21.3%) but not in adjacent matched normal gastric tissues. Overexpression of DARPP-32-STARD3 significantly increases gastric cancer cell proliferation *in vitro* and gastric tumor xenograft growth in nude mice. These effects are mediated by activation of the PI3K/AKT signaling pathway [47]. These findings strongly suggest that DARPP-32-STARD3 fusion transcript promotes tumorigenesis in gastric cancer. Another study included an investigation on the role of DARPP-32 in gastric tumorigenesis through regulation of CD44 splicing [48]. The findings indicated that DARPP-32 positively regulates the overall splicing activity, and specifically promotes the expression of the splice isoform, CD44E, in gastric cancer cells. Mechanistic studies revealed that DARPP-32 associates with SRp20 splicing factor in a protein complex, thereby stabilizing SRp20 protein and increasing its expression. Modulation of DARPP-32 expression indicated that DARPP-32 regulates the SRp20-dependent CD44E splicing and gastric tumor growth in a xenograft mouse model. Notably, the study indicated frequent overexpression of DARPP-32, SRp20 and CD44E proteins in human gastric primary tumors, further establishing an important role for DARPP-32 in gastric tumorigenesis [48]. In a recent report, the role of DARPP-32 and t-DARPP in breast tumorigenesis was investigated in a mouse mammary tumor model [49]. In contrast to DARPP-32, which was expressed in normal mouse mammary tissue and in some tumors, t-DARPP was exclusively expressed in tumors at equal or higher levels than DARPP-32, suggesting that t-DARPP plays a predominant role in breast tumorigenesis. Notably, knockout of the *DARPP-32* gene in MMTV (mouse mammary tumor virus promoter)-PyMT (polyoma middle T oncogene) transgenic mammary tumor mice reduced tumor growth, thereby demonstrating the important role of DARPP-32 proteins in breast tumorigenesis [49].

### DARPP-32 proteins in cancer cell survival and drug resistance

An initial report demonstrated that DARPP-32 proteins could protect cancer cells against drug-induced apoptosis. This was particularly evident in studies where both DARPP-32 and t-DARPP protected gastric cancer cells against camptothecin, sodium butyrate, and ceramide treatments in a p53-independent mechanism [39]. Expression of DARPP-32 and t-DARPP preserved the mitochondrial transmembrane potential in response to camptothecin, and this was associated with increased levels of Bcl2 protein [39]. Notably, the pro-survival function of both DARPP-32 and t-DARPP depends on Thr-75 phosphorylation residue [39]. A subsequent study indicated that the pro-survival function of t-DARPP involves increased AKT kinase activity and high levels of phospho-AKT (Ser-473) and phospho-GSK3β (Ser-9) proteins in gastric cancer cells [50]. In addition, the study demonstrated that t-DARPP activates CREB and ATF-1 transcription factors, which bind to a CREB response element (CRE) on Bcl2 promoter, in an AKT-dependent mechanism, leading to up-regulation of Bcl2 expression and increased cell survival in response to ceramide [50]. A recent report indicated that DARPP-32 binds Bcl2 and calcineurin (CaN) in a complex with inositol 1,4,5-triphosphate receptor (InsP3R, a ubiquitous intracellular Ca2+ channel), which induces a negative feedback loop that reduces Ca+2 release and apoptosis by decreasing phosphorylation of InsP3R in chronic lymphocytic leukemia cells [51]. The study concluded that this mechanism involving DARPP-32 might be exploited by Bcl2-overexpressing cancer cells to suppress apoptosis. On the other hand, one study found that DARPP-32 reversed the multidrug resistance (MDR) of Adriamycin (ADR)-resistant gastric adenocarcinoma cells (SGC7901/ADR), as these cells were significantly sensitized to vincristine, ADR, 5-fluorouacil, and cisplatin in response to DARPP-32 up-regulation [52]. Based on a single gastric cancer cell model, these controversial findings may suggest cell type specificity.

In a study, the role of DARPP-32 in mediating resistance to gefitinib, a small tyrosine kinase inhibitor specific for EGFR, was investigated in gastric cancer. The data demonstrated that DARPP-32 expression blocks gefitinib-induced apoptosis in gastric cancer cells. This is accompanied with activation of AKT, increased stability of the EGFR protein, and the colocalization of DARPP-32 with EGFR in a complex with ERBB3 on the cell membrane [53]. Mechanistic investigations concluded that DARPP-32 promotion of EGFR/ERBB3 protein interaction leads to EGFR phosphorylation and activation of PI3K-AKT signaling, thereby increasing cell survival and gefitinib resistance in gastric cancer cells [53]. In a report, DARPP-32 was implicated in mediating resistance to TRAIL (TNF-related apoptosis-inducing ligand) in gastric cancer. The data indicated that DARPP-32 expression suppresses TRAIL-induced apoptosis, accompanied with reduced cytochrome c release and activation of caspase-8, -9, and -3 in gastric cancer cells [54]. Further investigations indicated that DARPP-32 induces the expression of the pro-survival Bcl-xL protein through activation of Src/STAT3 signaling. In addition, DARPP-32 has been shown to prevent the TRAIL-induced caspase-dependent cleavage of NF-κBp65, thereby maintaining the activity of NF-κB activity and the expression of its target, the pro-survival FLIPs (FLICE-inhibitory protein). Together, the findings suggest that DARPP-32 promotes TRAIL resistance whereby it suppresses the intrinsic and extrinsic apoptosis pathways through regulation of Bcl-xL and FLIPs, respectively [54].

In a study, the role of t-DARPP in resistance to trastuzumab (Herceptin), a monoclonal antibody against ERBB2 receptor, was investigated in breast cancer [55]. The data indicated that t-DARPP expression was up-regulated in trastuzumab-resistant ERBB2-positive breast cancer cells. Genetic knockdown of t-DARPP sensitized these resistant cells to trastuzumab as indicated by increased activation of caspase-3 and apoptosis. Mechanistic studies demonstrated that t-DARPP was associated with the chaperone heat shock protein 90 (HSP90) and ERBB2 in a protein complex, suggesting that t-DARPP mediates trastuzumab resistance through regulation of ERBB2 receptor in trastuzumab-resistant breast cancer cells [55]. A subsequent study confirmed that t-DARPP overexpression was associated with AKT activation and trastuzumab resistance in breast cancer cells [56]. The findings indicated that t-DARPP overexpression and Thr-75 t-DARPP residue are essential for AKT activation and trastuzumab resistance in these cells. In addition, the study showed that DARPP-32 promotes resistance to trastuzumab, and overexpression of DARPP32 is associated with poor prognosis in breast cancer [56]. However, another study indicated that t-DARPP, but not DARPP-32, was overexpressed in a trastuzumab-resistant breast cancer cells, and demonstrated that t-DARPP overexpression was sufficient to confer resistance to trastuzumab and suppress trastuzumab-mediated dephosphorylation of AKT in sensitive breast cancer cells [42]. A recent study has shown that t-DARPP overexpression in HER2-positive breast cancer confers a survival advantage to cancer cells in response to lapatinib by suppressing lapatinib-induced BIM accumulation [40]. A related report indicated that t-DARPP promotes trastuzumab resistance through regulation of ERBB2 receptor in esophageal adenocarcinoma [57]. In fact, t-DARPP was shown to counteract trastuzumab-induced apoptosis, inhibit activation of caspase-3, and suppress trastuzumab-induced dephosphorylation of ERBB2 and AKT proteins. However, knockdown of endogenous t-DARPP reversed these effects and sensitized cells to trastuzumab. The findings from this study also indicated that t-DARPP associates with ERBB2, thus increasing its protein stability and interfering with the binding of trastuzumab with the ERBB2 receptor in esophageal adenocarcinoma cells [57].

### DARPP-32 proteins in cancer cell growth and proliferation

A study demonstrated mRNA overexpression of t-DARPP in 36% of breast cancers, predominantly observed in invasive ductal and intraductal carcinomas, as opposed to absent to very low t-DARPP expression in normal mammary tissue. Similarly, protein overexpression of DARPP-32/t-DARPP was observed in 35.5% of primary breast tumors, including invasive ductal carcinomas (43.7%) [41]. *In vitro* studies demonstrated that the overexpression of t-DARPP promotes cell proliferation associated with induction of phosphorylation of AKT (Ser-473) and its target GSK-3β (Ser-9) in breast cancer cells. The knockdown of endogenous t-DARPP or inhibition of PI3K reversed these effects, suggesting that the pro-growth function of t-DARPP depends on PI3K in breast cancer [41]. In a related study, the role of t-DARPP in regulating β-catenin signaling and proliferation was examined in upper gastrointestinal cancer cells [58]. The data indicated that the expression of t-DARPP increased cell proliferation and led to activation of β-catenin through phosphorylation of AKT (Ser-473) and GSK-3β (Ser-9) in a PI3K-dependent mechanism [58].

### DARPP-32 in cancer cell migration, invasion, and angiogenesis

**Figure 3:**
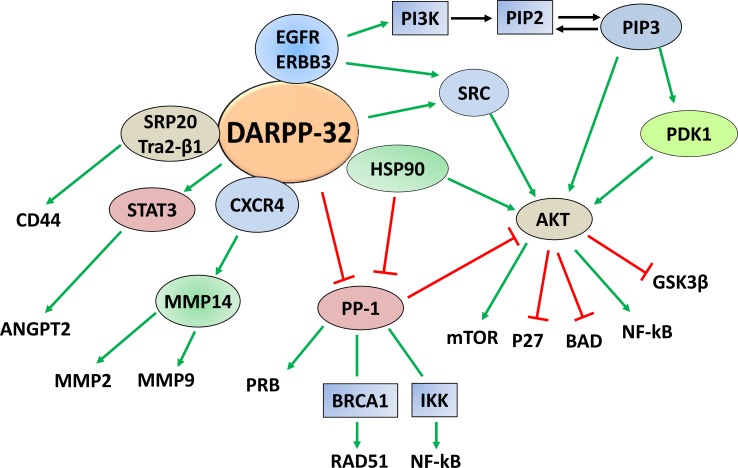
DARPP-32-regulated cancer signaling pathways Based on the published data, DARPP-32 constitutes a signaling hub that regulates multiple pathways important for carcinogenesis and tumor progression. Activation is depicted by green arrows and negative regulation is indicated by red T-lines.

A report indicated that overexpression of DARPP-32 in breast cancer cells that express DDR1, a receptor tyrosine kinase, significantly impaired cell migration. However, DARPP-32 expression in DDR1-deficient cells had no effect on migration [59]. It was demonstrated that DDR1 associates with DARPP-32 in a protein complex, which regulates migration, and the phosphorylation of Thr-34 residue is required for DARPP-32 function to impair migration of breast cancer cells. This was further supported using PKA inhibitors that abrogated the anti-migratory effect of DARPP-32 [59]. A related study investigated the role of the Wnt-5a signaling pathway in regulating DARPP-32-dependent inhibition of migration in breast cancer cells [60]. The study indicated that recombinant Wnt-5a induced cAMP elevation, leading to activation of PKA, phosphorylation of Thr-34-DARPP-32, and inhibition of cell migration. Further studies demonstrated that the inhibition of PP1 by phospho-Thr-34-DARPP-32 potentiated the Wnt-5a-mediated phosphorylation of CREB, a known PP1 substrate. Additionally, inhibition of the Wnt-5a-DARPP-32-CREB pathway, by expression of dominant negative CREB, reduced the anti-migratory effect of DARPP-32 in response to Wnt-5a [60]. On the other hand, another study showed that DARPP-32 overexpression promotes gastric cancer cell invasion [61]. The pro-invasive function of DARPP-32 was associated with an increase in the expression of the membrane-type 1 matrix metalloproteinase (MT1-MMP) and CXC-chemokine receptor 4 (CXCR4) proteins. Consistent with the role of MT1-MMP in activating MMPs, a substantial increase in MMP-2 activity was detected in DARPP-32-expressing cells. Inhibition or knockdown of CXCR4 suppressed DARPP-32-induced cell invasion. Further mechanistic studies showed that DARPP-32 and CXCR4 coexist in the same protein complex, leading to increased stability of CXCR4 protein because of reduced ubiquitination, following treatment with its ligand, CXCL12. These findings demonstrate that DARPP-32 enhances gastric cancer cell invasion by regulating CXCR4-mediated activation of the MT1-MMP/MMP2 pathway [61]. A prospective pilot study, using clinical and histopathological data of 100 patients with colorectal cancer between 1994 and 1997, revealed that DARPP-32 was highly expressed in patients with distant metastases, relative to patients without distant metastases. Further analysis indicated that DARPP-32 expression in primary colorectal tumors was a significant predictor of distant metastases, and could be a potential marker of poor prognosis [62]. A recent report indicated that DARPP-32 promotes angiogenesis through regulation of angiopoietin 2 (ANGPT2) in gastric cancer [63]. In fact, DARPP-32 or t-DARPP positively regulates mRNA and protein expression and secretion of ANGPT2 in gastric cancer cells, thereby inducing angiogenesis. Further mechanistic investigations confirmed the role of ANGPT2 in mediating DARPP-32-induced angiogenesis, and revealed that DARPP-32 proteins regulate ANGPT2 expression through activation of STAT3. Notably, DARPP-32 induces ANGPT2 expression in human gastric tumor epithelial cells, but not in tumor associated vascular endothelial cells [63]. A schematic diagram summarizing DARPP-32-regulated signaling pathways involved in tumorigenesis is shown in Figure 3.

## CONCLUSIONS AND PERSPECTIVES

**Figure 4:**
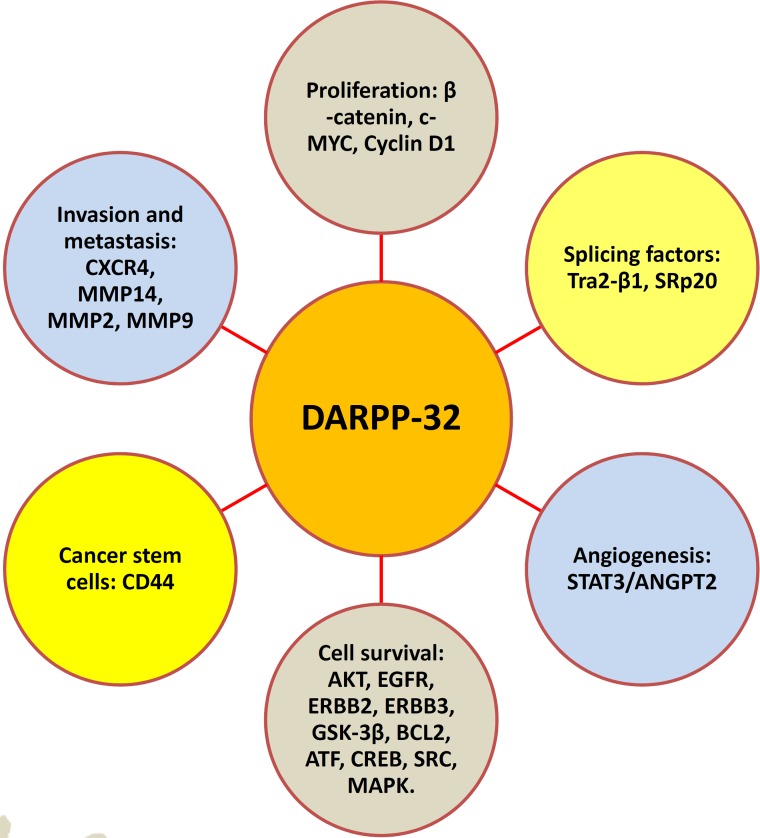
DARPP-32 regulates major hallmarks of tumorigenesis Accumulating published reports strongly suggest that DARPP-32 promotes cancer cell proliferation, survival, invasion and metastasis, and angiogenesis. Each DARPP-32-mediated function involves regulation of corresponding signaling pathways depicted in the schematic diagram.

A large body of research over the last three decades has established DARPP-32 as a major integrator of neurotransmission by dopamine and other neurotransmitters in response to drugs of abuse or psychostimulants. Additionally, several recent reports implicated DARPP-32 and t-DARPP proteins in CNS disorders and neurodegenerative diseases. The role of DARPP-32 proteins in tumorigenesis and drug resistance has been recently recognized and is a growing field of investigations that continue to unveil new functions of DARPP-32 (Figure 4). Collectively, the findings from the published reports strongly suggest that DARPP-32 proteins have potential as a biomarker of drug resistance or a therapeutic target in gastric cancer and other malignancies. In the era of personalized cancer medicine, it is imperative that future investigations and efforts align together for the development and testing specific pharmacological inhibitors that target DARPP-32 and its signaling pathways in preclinical and clinical settings.
